# Illustrations of the Difficulties Which Beset the Diagnosis of Some Cases of Disease

**Published:** 1850-11

**Authors:** Thomas Poyser

**Affiliations:** Fellow of the Royal College of Surgeons of England


*Illustrations of the Difficulties which beset the Diagnosis of some Cases of Disease.* By THOMAS POYSER, Esq., Fellow of the Royal College of Surgeons of England.—I have been much gratified by the perusal of a paper in the London Journal of Medicine for May, by Dr. Semple, on "Diseases of the Nervous System." The cases which he so candidly and graphically describes, are familiar to all who are in extensive practice, and show but too clearly, that in a great variety of cases which terminate fatally, the symptoms during life do not develope the true seat and nature of the malady. The great attention that has of late years been paid to general and morbid anatomy, and to pathology, with the mechanical aids that have been had recourse to, for the investigation of diseases, as well as the more patient and inductive method of studying them by those who practice medicine as a science, have no doubt tended materially to render diagnosis and symptomatology more clear and specific ; yet there is still a wide field open, in this most important department of medical practice, for future explorers.

To ascertain the true pathological cause of disease is confessedly the most difficult, as it is the most important part of medical practice. Indeed, as Celsus has well observed, "æstimatio causæ sæpe morbum solvit." Many causes, however, concur in practice, but more especially in the country, to render the patient observation of disease difficult, if not almost impracticable. Extensive practice, while it renders the practitioner familiar with the phenomena of disease and its terminations and best means of cure, leaves rarely much leisure for the niceties of physical and chemical exploration, required in obscure cases. They who have the most extensive field of practice, are apt, in the hurry of business, to depend on tact, a sort of intuitive gift, like the *tactus eruditus* of the surgeon : and hence their examination of cases is apt to become habitually superficial, and a routine mode of prescribing is also, from the same cause, too often allowed to usurp the place of rational treatment.

I do not state this to the disparagement of the general practitioners, than whom a more painstaking, intelligent or consciencious body of men does not exist, in our own or any other profession. But we cannot expect impossibilities; and the multiplicity and diversity of cases which the well-employed medical man has to visit daily, if spread over a great extent of country, forbid that minute attention and examination which are frequently required.

Another hindrance to the complete investigation of disease, when it terminates unfavorably in the country, is the great repugnance to *post-mortem* examinations. In large towns and public institutions this prejudice is not so strong; but in some rural districts, a request to have a *post-mortem* inquiry almost invariably gives offence to the relatives, and is very rarely permitted. The country practitioner has seldom, therefore, an opportunity of verifying his diagnosis by a *post-mortem* examination; and if he had doubts on his mind as to the cause and seat of the disease, during the life of his patient, those doubts have little chance of being cleared up by his death.

Occasionally, however, a post-mortem examination is allowed, and then we sometimes find that the true seat of the disease is revealed, which during life was not suspected, nor indicated by the symptoms. These unlooked-for terminations of disease are seldom given to the public; as they imply, (though often unjustly,) a want of skill, or perspicacity, in the medical attendant: while periodical works teem with cases terminating favorably, or where the examination after death proves the diagnostic skill of the narrator. In systematic treatises, too, and monographs on particular diseases, the general history of the symptoms, treatment, and termination of the ailment, proceeds too smoothly to meet the exigencies and stern realities of actual practice. Untoward and unlooked for terminations of disease are seldom adverted to, or recorded; and yet these furnish the most important and instructive part of the history, as they are beacons or landmarks for future inquiries.

In the short but elegant preface to the transactions of the College of Physicians, written, as is generally supposed, by the late Dr. Heberden, is this paragraph: "It is to be wished, that writers would not confine themselves to relate only their successful practice. A physician of great experience might write a very useful paper, if he would have the courage to give an account of such methods of cure only, as he had found to be ineffectual or hurtful." With the wish to further a suggestion coming from such high authority, I published a few years ago, in the fifth volume of the Transactions of the Provincial Medical and Surgical Association, some cases and dissections "to show the uncertainty and difficulty of diagnosis; from the symptoms during life not being indicative of, or bearing any proportion to, the extent of morbid lesion discoverable after death." The cases to be offered to the readers of the London Journal of Medicine, may be regarded as a continuation of these reports. Having no system to establish, or theory to support, they will be selected indiscriminately, from notes taken at the time: and although, perhaps, but little may be learned from them in the way of practice, yet

I trust that, from the reasons above stated, they will not be found uninteresting or useless. An important object will be answered by their publication, if it induce others, whose opportunities of observation from public hospitals and other sources are much greater than mine, to publish such results of their experience, as may illustrate the difficulties which beset the diagonsis of some cases of disease.

Case i. Obscure Neuralgic Pains: Disease of the Brain, and Enlargement of the Liver. Mrs. —, aged 40, the mother of a large family, and a lady of great intellectual attainments, who had usually been in the enjoyment of good health, was attacked in the Autumn of 1844 with acute pain in the right groin, or rather in the inner and upper margin of the ilium. The pain was limited in extent, and might be covered, as she expressed it, with her finger, but was so acute as to deprive her almost entirely of sleep, and to render any motion of the part nearly insupportable. There was no swelling, redness, nor any appearance of inflammation. This pain continued some months, without varying its seat or character, and but little controlled by treatment.

It was considered to be neuralgic; and every means that could be suggested, both constitutional and local, were tried by an eminent surgeon who then attended the case. During the progress of this affection, Mrs. — herself discovered, while in bed, (to which she had long been confined by the neuralgic pain,) a swelling in the right side below the liver. On examination, this viscus was found much enlarged, extending four or five inches below its normal size and situation, of a stony hardness; but unattended with pain or tenderness on pressure. It seemed to account for the pelvic pain, inasmuch as the pressure from this indurated mass on the nerves might occasion it. Mercury with taraxacum, and iodine and mercurial frictions, were therefore had recourse to, without any alleviation of pain, or diminution of the hepatic disease.

In the Spring of 1845, the patient was removed to her mother's house in this neighborhood, and on April 5th I saw her. In addition to the enlargement of the liver, and the acute pain on the inner margin of the crista ilii, there was now tympanitis, the bowels being enormously distended with flatus. There was no pain in the head, no fever, and the pulse and tongue were natural: but there was a degree of irritability of mind and fretfulness which was remarkable, as Mrs. —'s natural disposition was peculiarly placid and gentle. It is unnecessary to detail the treatment, as but little or no relief was obtained from it.

The tinctura cannabis indicæ soothed and tranquillized the nervous feelings; and the unguentum aconiti as a local application, (suggested by Sir Benjamin Brodie, who was consulted,) in some degree assuaged the pain; but the general character of the symptoms continued unabated; till about the middle of April, when the pain began to diminish, and, in two or three days, went off altogether. After the removal of the pain, Mrs. —'s spirit and appetite improved; she was able to sit up and walk about her room; and although the tympanitic swelling and enlargement of the liver did not diminish, yet she was cheered by the freedom from pain, and the prospect of recovery.

On the 28th of April, while sitting in her chair, Mrs. — had an apoplectic seizure, which deprived her of speech and the power of swallowing, from which she never rallied. She expired May 3rd.

*A post-mortem examination* was made thirty-six hours after death. On removing the calvarium, the external part of the brain presented no diseased appearance; but, on cutting into the left hemisphere, it was found to be quite degenerated in structure. The whole of the anterior lobe was converted into a thick purulent fluid; and, in the middle of this large abscess, there was a clot of blood of the size of a walnut. This coagulum appeared to be of recent formation. The left middle lobe was pulpy and diseased, but the other parts of the cerebrum and cerebellum had a firm consistence and healthy structure. The abdomen was very large, from the bowels being enormously distended with flatus, but there was no extravasated fluid or gas in the cavity. The left lobe of the liver presented a very diseased appearance. It was enlarged to more than twice its usual size, had almost a stony hardness, was mottled and tuberculated externally, and on cutting into it, its texture was gristly and exhibited that diseased structure termed mammary sarcoma. The inner edge of the ilium, where the pain had existed so many months, was carefully examined, but there was no morbid appearance discoverable in the part, or in the nerves leading to it. The thorax was not opened.

Remarks. This case is, I think, interesting, and worthy to be recorded. It shows that the symptoms did not point to the true seat of disease, till a short time before the patient's death; and that extensive disorganization may be going on in the brain, without impairing its functions, or manifesting those signs by which it may be detected. The fretful and altered manner of the patient led to the apprehension that softening of the brain might be going on; but the total absence of pain in the head, and of rigors, or any indication of inflammatory action, rendered the diagnosis very difficult. It is highly probable, that the pain in the right side of the pelvis was occasioned by disease of the left side of the brain, and furnishes an additional instance to those recorded by Sir Henry Halford, where neuralgia was the effect of cerebral disorganization. When the latter disease had so far advanced as to disqualify the nerves from suffering so exquisitely, then the pain ceased. The case shows the importance of attending to the brain, in all long-continued and obscure neuralgic pains, although the functions of that organ may not be impaired.—*London Journal of Medicine.*

